# The Comparative Study of the Therapeutic Effects and Mechanism of Baicalin, Baicalein, and Their Combination on Ulcerative Colitis Rat

**DOI:** 10.3389/fphar.2019.01466

**Published:** 2019-12-13

**Authors:** Shuai Liang, Xin Deng, Lei Lei, Yao Zheng, Jiao Ai, Linlin Chen, Hui Xiong, Zhinan Mei, Yung-Chi Cheng, Yongshen Ren

**Affiliations:** ^1^School of Pharmaceutical Science, South-Central University for Nationalities, Wuhan, China; ^2^School of Pharmacy, Hubei University of Chinese Medicine, Wuhan, China; ^3^School of Medicine, Yale University, New Haven, CT, United States

**Keywords:** baicalin, baicalein and combination, ulcerative colitis, young *Scutellaria baicalensis*, withered *Scutellaria baicalensis*, metabolism distribution, therapeutic effects, mechanisms comparison

## Abstract

**Introduction:** Ulcerative colitis (UC) is an inflammatory bowel disease with a high incidence rate and a difficult treatment regimen. Recently, significant advances in the treatment of intestinal diseases, particularly UC, have been made with the use of the drugs baicalin and baicalein, separately or in combination. However, the therapeutic efficacy and mechanism of action of baicalin, baicalein, and their combination therapy, in the treatment of UC has not been fully elucidated.

**Materials and Methods:** we constructed a UC rat model that encompassed a variety of complex factors, including a high-sugar and high-fat diet, a high temperature and humidity environment (HTHE), excess drinking, and infection of *Escherichia coli*. Model rats were then treated with baicalin, baicalein, or a combination of the two.

**Results:** The results showed significant differences in the therapeutic effects of baicalin, baicalein, and the combination therapy, in the treatment of UC, as well as differences in the inhibition of inflammation *via* the nuclear factor-κB and MAPK pathways. The rat model of UC was established as described above. Then, the rats were treated for 7 days with baicalin (100 mg kg^-1^), baicalein (100 mg kg^-1^), or both (100 mg kg^-1^, baicalin: baicalein = 4:1/1:1). Clinical symptoms and signs, body temperature, organ indices, histopathology, blood biochemistry, and metabolites were examined to compare treatment effects and indicators of UC. Baicalin, YSR (Young *Scutellaria baicalensis* ratio of baicalin and baicalein), baicalein, and WSR (Withered *Scutellaria baicalensis* ratio of baicalin and baicalein) had significantly different effects in terms of clinical symptoms and signs, body temperature, organ indices, serum inflammatory cytokine levels, blood biochemistry, and histopathology changes in the main organs; YSR exhibited the best treatment effects. LC-MS/MS was used to detect the conversion of baicalin, baicalein, or both, into the six types of metabolites: baicalin, wogonoside, oroxin A, baicalein, wogonin, and oroxylin A. The levels of the six metabolites under the different treatment conditions were significantly different in the large intestine, small intestine, and lungs, but not in the blood. The levels of the six metabolites were significantly different in the large intestine, small intestine, and lung, but not in the serum.

**Conclusion:** All these results indicate that baicalin and baicalein should be used more accurately in specific diseases, especially baicalin or high content of baicalin in *Scutellaria baicalensis* (Tiaoqin) should be preferred in treatment of UC.

## Introduction

Ulcerative colitis (UC) is a common refractory and life-long disease (Sedghi, 2016; Murakami et al., 2018). The common presenting symptoms include gastroenteritis, fever, diarrhea, rectal bleeding, mucus in stools, sense of urgency for bowel movements, and abdominal pain (Xu et al., 2016). The occasional extraintestinal manifestations include arthritis, iridocyclitis, episcleritis, erythema nodosum, liver dysfunction, and skin lesions. The etiology of UC remains unknown. Modern researchers believe that the etiology involves genetic factors, environmental factors, microbial infections, and other factors (Dulai and Jairath, 2018). Some researchers consider UC to be an autoimmune disease (Wiese et al., 2016; Kim et al., 2017).

Currently, UC cannot be completely cured and life-long treatment is needed in most cases (Nowarski et al., 2015). Further, 20–30% of patients with severe UC require surgery as a part of their treatment (Lanzoni et al., 2008). Anti-inflammatory treatment with drugs such as antibiotics, aminosalicylic acid, and glucocorticoids, as well as immunosuppression treatment *via* hormone therapy, are the primary treatment approaches. However, adverse side-effects and limited efficacy have been reported (Ishiguro et al., 2006). There is also doubt as to whether hormones should be used continuously in the chronic phase of UC (Caprilli et al., 2007). Thus, more rational treatment of this disease is needed.

It is believed that abnormality of the intestinal mucosal immune system plays an important role in the pathogenesis of UC. Diffuse inflammation of the intestinal mucosa and crypt abscesses are the typical symptoms observed during the active period of UC. When crypt abscesses collapse, the mucosa appears presents with a large number of small ulcers that gradually merge into large ulcers. These large ulcers continue to destroy the intestinal mucosa causing tissue damage, and can even result in the formation of cancerous lesions (Xavier and Podolsky, 2007). The use of anti-inflammatory and immunosuppressive drugs for treatment of UC results in a cycle of remission and recurrence (Sha et al., 2013).

**Figure 1:**
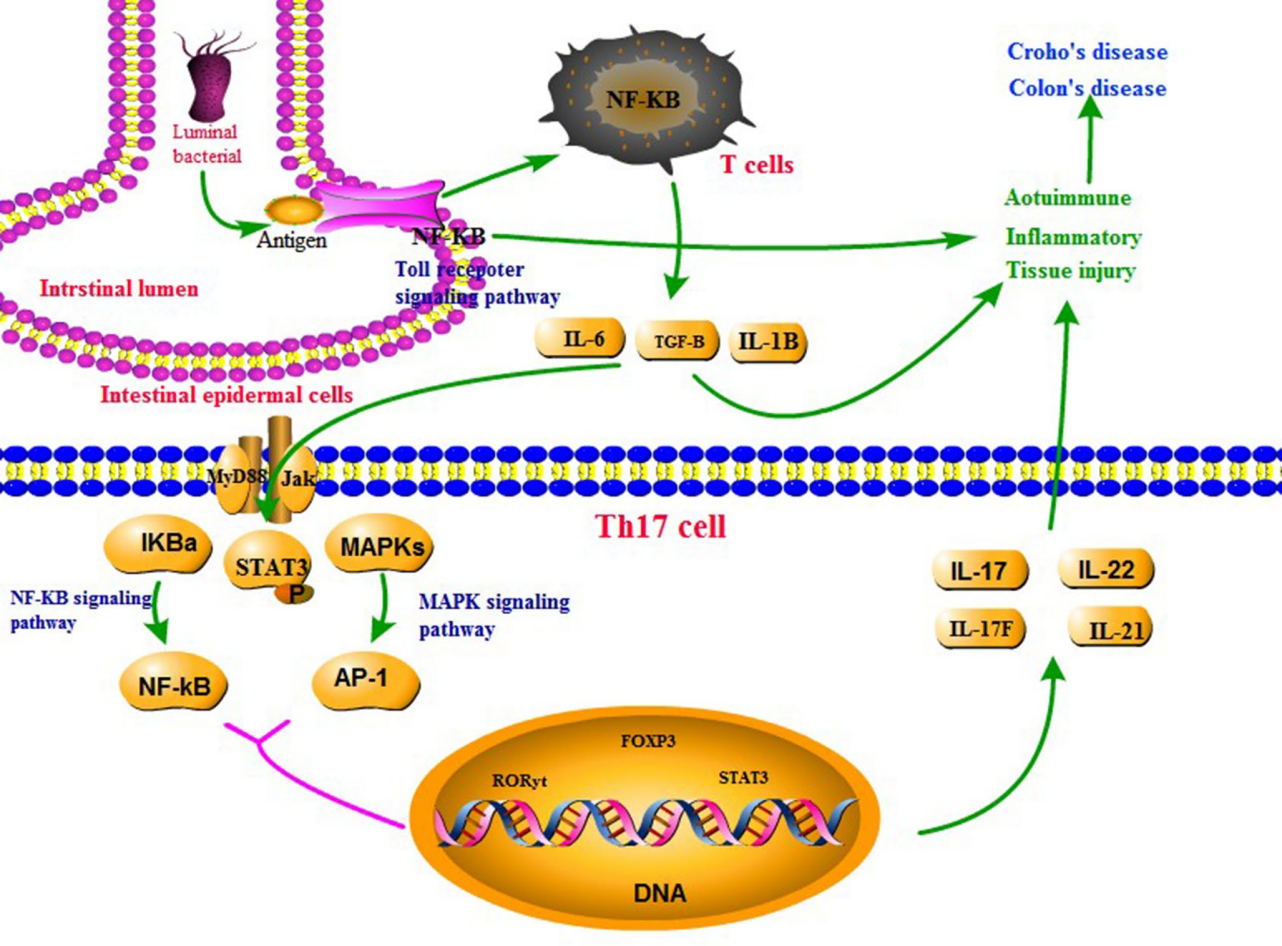
The mechanism of UC.

Th17 cells are a subset of Th cells that produce interleukin (IL)-17. These cells are involved in the development and progression of many inflammatory and autoimmune diseases (Machino-Ohtsuka et al., 2014). Recently, scientists have discovered the proliferation of Th17 T helper cells (Th17) in UC, and this appears to be of great significance to autoimmune diseases and the body’s defense response (Kuwabara et al., 2017). TH17 primarily acts against extracellular bacteria and mold. Normally, the differentiation of TH17 helper cells is induced by IL-6 and transforming growth factor (TGF)-β, and is followed by the release of cytokines such as IL-1β, IL-6, and tumor necrosis factor (TNF)-α against extracellular bacteria and mold immune responses (Afzali et al., 2007). The main transcription factors of TH17 are STAT3 and RORγ. IL-17 and TNF-α secreted by CD4 T cells can activate neutrophils to phagocytize and digest extracellular bacteria and fungi (Ivanov et al., 2006). In addition, IL-6 can activate a complement reaction and directly kill extracellular bacteria and fungi. A balance of Treg/Teffs cells is necessary to support immune homeostasis and prevent autoimmunity. Treg cells have immunosupressive function in autoimmune disorders; they can control expansion and activation of T helper cells and can maintain self-tolerance. Further, multiple studies suggest that subsets of Tregs are important in the host’s resistance to infectious diseases. Teffs, Th1, and Th17 are pro-inflammatory T cells that contribute to the development of autoimmune processes (Li et al., 2017), as shown in **Figure 1**.

At present, aminosalicylic acid, glucocorticoids, and immunosuppressive agents are commonly used for the treatment of UC in clinical practice; incremental drug therapy strategies are also often used. Aminosalicylic acid is still the mainstay of UC treatment. Glucocorticoids have strong anti-inflammatory effects and rapid induction, but given their adverse side effects and hormone dependence or resistance, their use must be strictly controlled in clinical practice (Lanzoni et al., 2008).

Given the difficulty in treating UC, it is important to look for alternative therapeutic strategies. Traditional Chinese Medicines (TCMs) such as *Scutellaria baicalensis* have been widely used to treat UC. These medicines have multi-component and multi-target characteristics. In recent years, a large number of clinical and experimental studies have shown that TCM can regulate the Th17/Treg balance, which is an important mechanism in the treatment of UC (Fan et al., 2009). Previous studies have shown that baicalin and baicalein, the two major components of *Scutellaria baicalensis* exert similar pharmacological activities, including anti-inflammatory, anti-oxidation, anti-bacterial, and liver-protection effects. However, recent studies have indicated that the antibacterial, anti-inflammatory (Zhang et al., 2013), anti-oxidation (Gao et al., 2001) and liver-protection (Huang et al., 2012) effects of baicalin and baicalein are significantly different (Liang et al., 2018). The mechanisms underlying their different therapeutic effects remain unknown.

**Figure 2:**
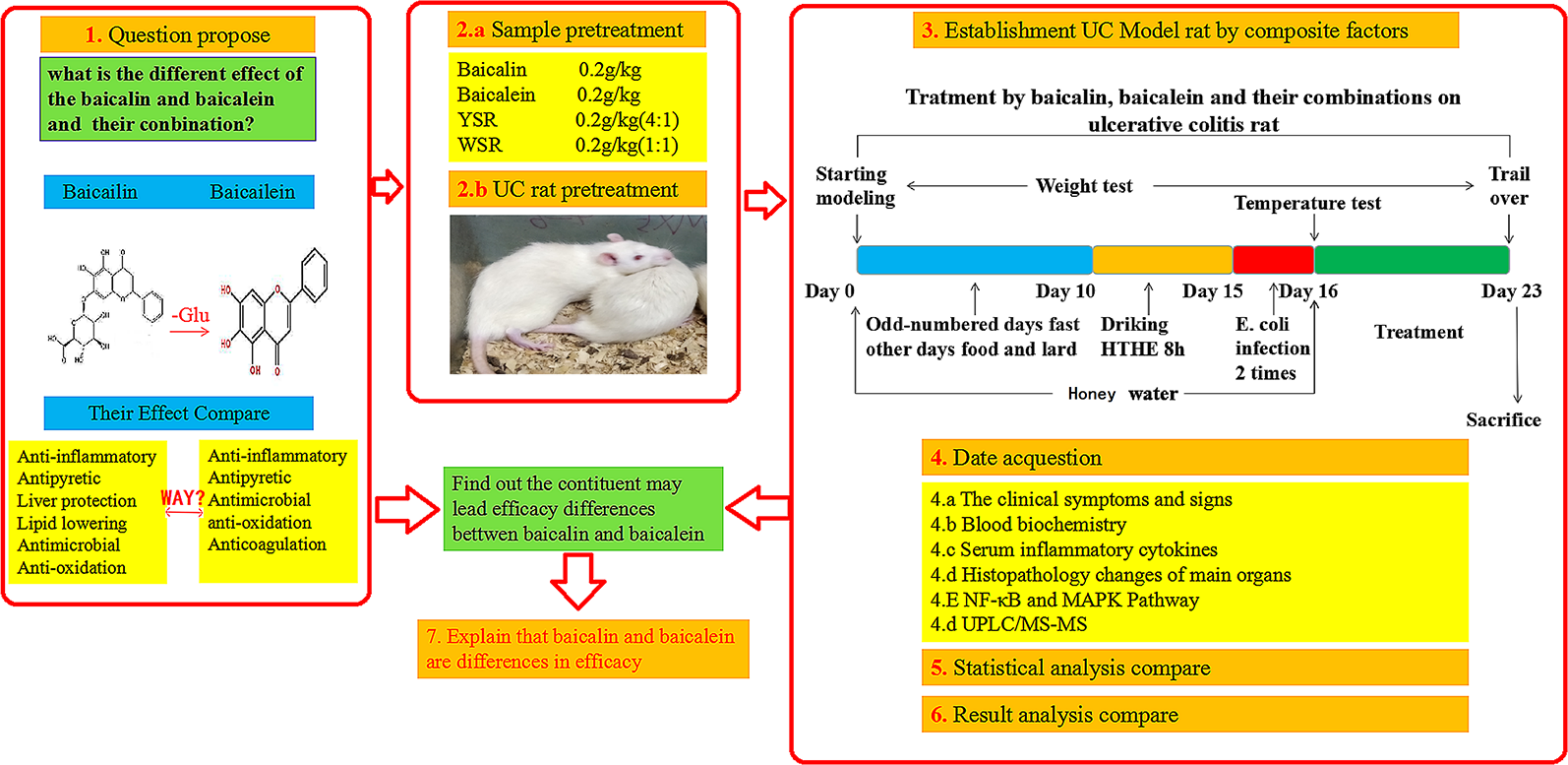
Experiment flowchart.

Therefore, in this work, we developed a UC rat model to study the differences in the effects of baicalin, baicalein, or a combination of the two, with respect to pharmacodynamics, metabolism, and underlying mechanisms. This study provides accurate and reliable evidence to evaluate the usage of baicalin and/or baicalein for the treatment of UC. Moreover, we reveal the possible mechanism underlying the effects of multi-ingredient herbal medicines for the treatment of UC. As shown in **Figure 2**.

## Experimental Method

### Materials and Reagents

Baicalin (CAS number 21967-41-9), baicalein (CAS number 491-67-8), and sodium hydroxymethylcellulose (CMC-Na; CAS number 9085-26-1) were purchased from Sinopharm Co., Ltd. Pentabarbital (CAS number 57-33-0), alanine aminotransferase (ALT), aspartate aminotransferase (AST), γ-glutamyl transpeptidase (GGT), superoxide dismutase (SOD), malondialdehyde (MDA), nitric oxide (NO), triglyceride (TG), cyclic guanosine monophosphate (cGMP), cyclic adenosine monophosphate (cAMP), sodium-potassium adenosine triphosphatase (Na^+^K^+^-ATPase), interleukin-1β (IL-1β), interleukin-17 (IL-17), and interleukin-6 (IL-6) kits were provided by Nanjing Jiancheng Bioengineering Institute. Sulfasalazine, Red Star Erguotou liquor, and honey were purchased from Jingdong e-market. Enterotoxigenic *Escherichia coli* (*E. coli*; No.: ATCC 25922) was provided by the National Institutes for Food and Drug Control of China. The nutrient agar was obtained from Qingdao Hope Bio-Technology Co., Ltd. (Qingdao, China). All reagents used were analytical grade.

### Preparation of Drug Suspension Solution

Baicalin, baicalein, and sulfasalazine were diluted in CMC-Na and the following suspension solutions were constructed: baicalin (100 mg kg^-1^), baicalein (100 mg kg^-1^), young *Scutellaria baicalensis* with a 4:1 ratio of baicalin to baicalein (YSR; 100 mg kg^-1^), withered *Scutellaria baicalensis* with a 1:1 ratio of baicalin to baicalein (WSR; 100 mg kg^-1^), and sulfasalazine (100 mg kg^-1^). Solutions were stored in the dark at 4 °C and were used within 5 days of preparation. The proportional relationships between baicalin and baicalein in the YSR and WSR solutions were determined based on a preliminary study of the ratios of baicalin and baicalein in young (1–3 years) *Scutellaria baicalensis* (Tiaoqin in Chinese) and withered (above 3 years) *Scutellaria baicalensis* (Kuqin in Chinese).

### Experimental Animals and Grouping

Fifty-six Sprague-Dawley (SD) rats (180–220 g) were housed in an SPF-level laboratory. The room temperature was regulated at 18–25°C with 50 ± 5% humidity. A 12 h light/dark cycle was implemented and the rats were fed a standard diet and given free access to water. Animal welfare was ensured and experimental procedures were performed in accordance with the Guide for the Care and Use of Laboratory Animals (Ministry of Science and Technology of China, 2010). This study was approved by the Animal Ethics Committee and Institutional Animal Care and Use Committee of South-Central University. After acclimatization for 7 days, the rats were randomly divided into eight groups with 7 rats in each group: normal control group (NC group), UC model group (UC group), self-healing group (self-healing group), baicalin group (baicalin group), baicalein group (baicalein group), YSR group (YSR), and WSR group (WSR). We chose the dose of baicalin according to the clinical dose, which was converted into an equivalent dosage.

### *In Vivo* Experimental Protocol

In the model, self-healing, baicalin, baicalein, YSR, and WSR groups, the rats were given free access to honey water (30%) throughout the modeling process. In the first 10 days, the rats were fasted and fed sufficient food combined with a lard gavage (4 ml/200 g weight at 37°C) on alternate days. Afterward, rats were gavaged with liquor (56° Red Star Erguotou, 2 ml/200 g body weight) every morning. Then, the rats were placed in a humid and hot climate chamber at 33 ± 2°C with 93 ± 2% humidity (HTHE) for 8 h, once daily for 5 consecutive days. Finally, the rats were gavaged twice (1 ml/100 g and 0.5 ml/100 g, respectively) with 1.6×10^9^ CFU/ml E. coli suspensions (prepared by activating the bacteria, shake-flask culturing, expanding, and propagating) spaced 24 h apart. In the NC group, the rats were fed a standard diet under natural conditions and were gavaged with an equal volume of normal saline. In contrast, 1 h after the second administration of *E. coli*, the baicalin, baicalein, YSR, and WSR groups were gavaged with the respective drugs (100 mg/kg/day, respectively) once daily for 7 consecutive days, with a volume corresponding to the volume of normal saline administered to the UC and self-healing groups. After the experiment, all rats were anesthetized intraperitoneally with 1% pentobarbital sodium. Blood samples were collected from the abdominal aorta using EDTA-K_2_ anticoagulant and non-anticoagulant vacuum blood collection tubes. Following this, the rats were sacrificed and tissue samples were quickly obtained from the ileum and colon (both samples were 1 cm long starting from the ileocecal orifice) and were fixed in 10% neutral formalin.

### Observation of the General State of Rats

All rats were observed for changes in their mental and active state, posture, hair, appetite, crissum, stools, urine, and tongue. Body weights were also recorded every day during the experiment.

### Body Temperature Measurement

The anal temperature of each rat (thermometer gently inserted 2 cm into the anus of the rat) was measured with a digital thermometer for 48 h after the first drug administration. In the first 12 h, temperatures were measured every 0.5 h, and thereafter, temperatures were measured every 4 h until 48 h had passed.

### Determination of Physiological Indices

Tissue samples from the heart, lung, liver, spleen, kidney, brain, small intestine, and large intestine were weighed and the corresponding organ indices (mg/g) were calculated. Further, 72 h after drug administration, stools and urine (for examination of urinary metabolites) were collected for 12 h.

### Blood Biochemical Assay

After standing for 2–4 h at 4°C, the blood samples (from non-anticoagulant vacuum blood collection tubes) were centrifuged at 3000 rpm for 10 min. Serum samples were separated and stored at -80°C for further use. Serum biochemical indices, including ALT, AST, GGT, NO, SOD, MDA, ATP, and TG, were then detected.

### Determination of Cytokines

Serum samples stored at -80°C were thawed at 4°C prior to analysis. IL-1β, IL-6, IL-17, cAMP, and cGMP tests were performed according to the manufacturers’ instructions.

### Histological Staining

Colon and ileum tissue samples were cut into 1 cm slices and were fixed with 10% formaldehyde solution for more than 24 h. The samples were then washed with running water to remove the formaldehyde, dehydrated with ethanol, made transparent with xylene, embedded in paraffin, sliced (4 µm), stained with hematoxylin and eosin (H&E), and then observed under a microscope.

### Western Blotting

Colon tissues were first ground in liquid nitrogen. The cells were then washed with PBS three times before they were lysed in RIPA buffer (Thermo, Waltham, MA, USA) containing a protease and phosphataseinhibitor cocktail (Roche Ltd., Basel, Switzerland). Total protein was collected according to the manufacturer’s instructions. The protein concentration was measured using the Bio-Rad assay system (Bio-Rad, Hercules, CA, USA). For Western blotting analysis, protein extracts (40 µg) were separated by electrophoresis on 10% sodium dodecyl sulfate-polyacrylamide gels and were then transferred to polyvinylidene fluoride membranes (PVDF membranes; Millipore, Darmstadt, Germany). After blocking with 5% skim milk in PBS for 1.5 h, the proteins were incubated with relevant anti-bodies at 4°C overnight, washed three times with PBS, and then incubated with corresponding secondary antibodies at room temperature for 1  h. Specific proteins were detected by an Odyssey™ Infrared Imaging System (Gene Company, Li-Cor, USA).

### LC-MS/MS Quantitative Determination

#### Lung, Small Intestine, Colon, and Serum Samples

All samples were weighed on an electronic balance (AL204, Mettler Toledo) and were homogenized in two volumes of cold purified water using an electric homogenizer (IKA T10, Germany) in an ice bath. Then, 500 µL of homogenate was placed in an Eppendorf tube and 1500 µL of 50% methanol and 50% acetonitrile (v/v = 1:  1) solution containing 0.1% formic acid was added; the solution was then vortexed for 3 min. The precipitate and the supernatant were then separated by centrifugation at 20 000 (×*g*) for 20 min at 4°C. The supernatant (1500 µL) was evaporated to dry under a stream of nitrogen gas at 37°C and was then re-suspended in 200 µL of methanol. The mixture was vortexed well and centrifuged at 20 000 (×*g*) for 10 min at 4°C. Then, 150 µL of the supernatant was transferred to an autosampler vial (2 ml; Agilent) with a vial insert (250 µL insert, polypropylene, Agilent) and a 5 µL aliquot was injected into the LC/MS-MS system for analysis.

#### Analysis Method

##### UPLC conditions

Chromatographic separation was performed in 9 min gradients over an Eclipse Plus C_18_ column (RRHD 2.1 mm × 50 mm, 1.8 µm). A flow rate of 0.3 ml min^-1^ and a column temperature of 35°C were set for separation of biological samples. The mobile phase (A) consisted of acetonitrile while (B) was water (containing 0.1% formic acid). The step gradient program was as follows: 22% (v/v) B at 0–6 min, 45–0% B at 6–7 min, 0% B at 7–8 min, and 0–78% B at 8-9 min. The re-equilibration time of gradient elution was 1 min and the injection volume was 5 µL.

##### MS conditions

LC/MS analysis was performed using a Q Exactive mass spectrometer (Thermo Fisher Scientific, USA). A heated electrospray ion source (HESI) was used for ionization. The HESI parameters were optimized as follows: sheath gas flow rate 35 units, auxiliary (aux.) gas flow rate 10 units, auxiliary gas heater temperature 350°C, gas flow 9 L/min, spray voltage 4 kV for (-)-ESI and 3.5 kV for (+)-ESI, and S lens RF level 50.

### Statistical Analysis

All data are expressed as mean ± S.D. (n = 7). Differences among groups were tested with one-way analysis of variance (ANOVA) followed by Duncan’s multiple range test. Analyses were performed with IBM SPSS V.23.0 (SPSS Inc., Chicago, USA). Significant differences were set at P < 0.05.

## Results

### Changes in Clinical Symptoms and Signs

Before the experiment, all rats were active and their fur was smooth, white, and clean. Their diets, stools, and urine were all normal. At the beginning of the experiment, the rats in the NC group exhibited no obvious abnormalities and there was a gradual increase in their weights (Yi et al., 2010).

In all other groups, during the initial 10 days of feeding a high-sugar and high-fat diet, all rats exhibited increased water intake and urination. The urine was yellowish on the fasting days, while the amount of the urine decreased on the feeding days. The stools were loose from the 6th to 8th day, and then became dry and yellow. The rats’ weights increased on the feeding days and decreased on fasting days; however, the overall trends were upwards. During the 5 days of exposure to the HTHE conditions, their weights gradually decreased, their stools were sticky and yellow, and some rats exhibited loose stools. When the rats were placed in the hot chamber, anal temperatures were increased and they showed depression, decreased appetite, yellow-tinted and tangled fur, and a flabby and droopy scrotum. During the challenge with Escherichia coli stage of the experiment, the rats exhibited diarrhea and higher anal temperatures. Their stools were loose, sticky, yellow and smelly, and some exhibited bloody mucopurulent discharge (Zhong et al., 2019).

Twenty-four hours after drug administration, the rats in each experimental group were more active than those in the model group; there was a gradual increase in their appetite and activity. Two-three days after drug administration, their symptoms decreased significantly. Compared with the model group, their fur was smooth and shiny, their diarrhea was relieved, and their body temperature returned to normal. Five days after drug adminis­tration, the UC rats returned to a normal state. Their stools were dry, their urine was transparent, they were mentally active, and their body weights gradually increased. The results show that the UC model was successfully induced and the disease symptoms were improved by all drugs. YSR exhibited the best treatment effects.

### Temperature Changes in the 48 H After Drug Administration

**Figure 3:**
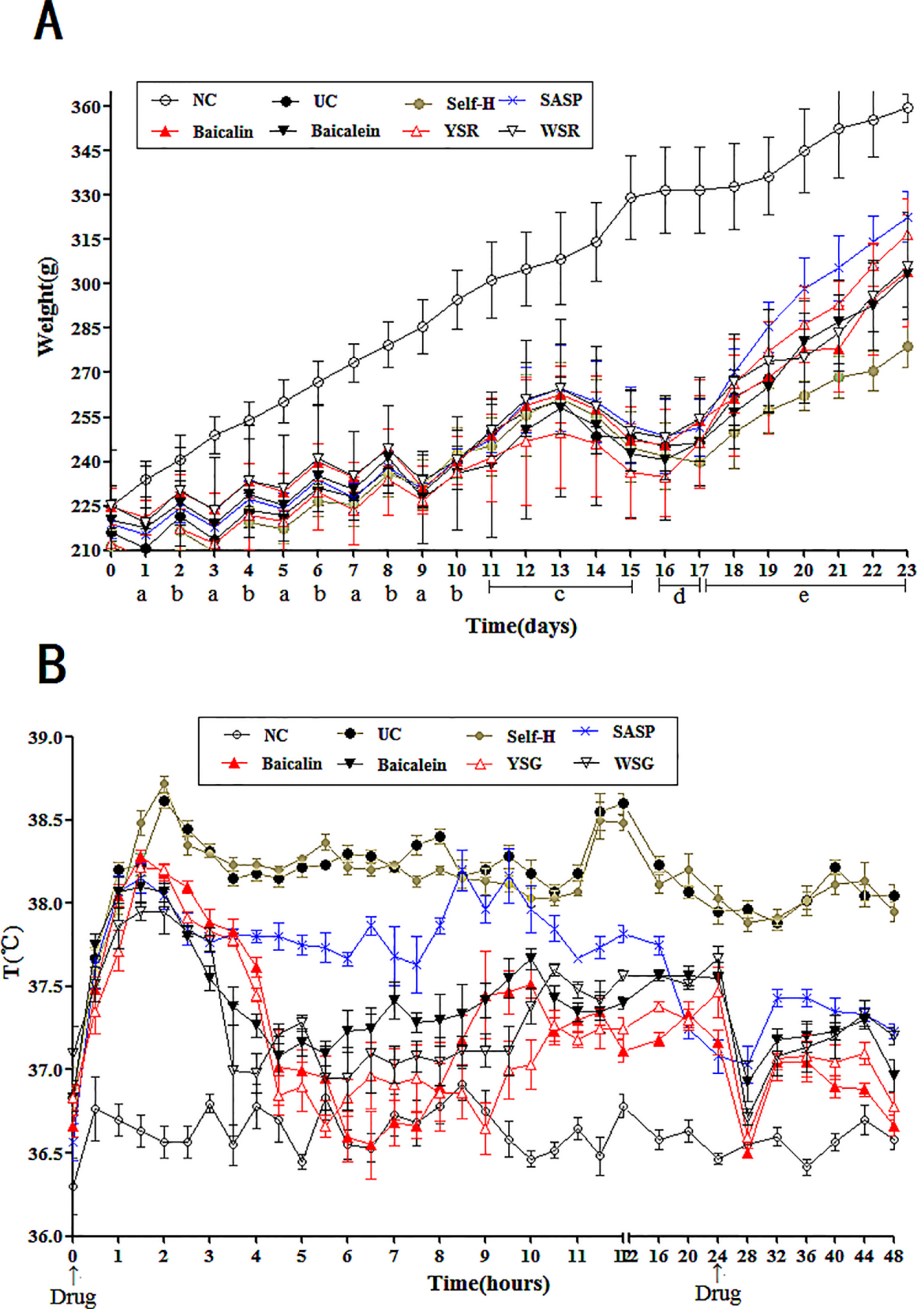
Changes of body temperature and body weight in rats. **(A)** Changes of body weight in rats; a, fast; b, diet with lard; c, drinking Erguotou with HTHE; d, *E coli.* infection; e, treatment. **(B)**, Changes of 48-h body temperature in rats. The data are presented as the mean ± S.D. (n = 7).

As shown in **Figure 3B**, within 48 h of the first drug dose, the average body temperature of the NC group fluctuated around 36.6°C. Compared with the NC group, the body temperature of the model group was significantly increased (P < 0.05). The model group reached a maximum temperature of 38.9°C before stabilization of temperature at 38.3°C. Compared with the model group, the average body temperatures of the drug groups were significantly reduced to different degrees (P < 0.01). The results indicate that the UC model resulted in a long-term increase in body temperature, and this body temperature increase could be reversed by drug administration. In particular, baicalin and YSR had the best efficacy. When considering all indices of body temperature, baicalin was more effective than baicalein, and YSR was more effective thanWSR, indicating that baicalin is superior at regulating body fever.

### Organ Indices

As shown in **Table 1**, the tissues and organs of the NC group were observed to be normal. The livers, spleens, lungs, and kidneys of the model group and the self-healing group had different degrees of congestion, edema, degeneration, necrosis, and inflammation. The organ indices for each organ in the model group was significantly higher than in the NC group (P < 0.05). The organ indices of the treatment groups were significantly lower than those of the UC group. The rats in the UC group exhibited widespread abnormal organ morphology, including abnormal morphology of the spleen, lungs, liver, and brain. Body edema and inflammation were evident in the UC model. The spleen and liver of the baicalin group were healthier than the baicalein group, and the YSR group exhibited better organ morphology than the WSR group. This indicates that baicalin has more potency for regulating the spleen and liver indices. However, for the lungs and brain, baicalein was superior to baicalin, and WSR was superior to YSR, indicating that baicalein has more potency for regulating the lungs and brain.

**Table 1:** The origin indices in rats.

Group	Spleen (mg/g)	Heart (mg/g)	Lung (mg/g)	Liver (mg/g)	Kidney (mg/g)	Brain (mg/g)
**Normal control (NC)**	1.79 ± 0.09	3.67 ± 0.04	3.96 ± 0.04	29.96 ± 2.11	7.03 ± 0.04	5.60 ± 0.04
**Model control (UC)**	4.60 ± 0.18**	3.90 ± 0.81	6.11 ± 0.14**	43.74 ± 4.28**	7.59 ± 0.25*	7.11 ± 0.84**
**Self-healing control**	4.15 ± 0.16**	3.80 ± 0.41	5.86 ± 0.09**	39.88 ± 1.42**	7.37 ± 0.25	6.65 ± 0.36*
**Sulfasalazine (SASP)**	2.90 ± 0.25^#^	3.72 ± 0.16	4.58 ± 0.04^##^	35.45 ± 4.36^#^	7.12 ± 0.49	6.31 ± 0.25^##^
**Baicalin**	2.37 ± 0.04^##^	3.62 ± 0.04	5.10 ± 0.37^#^	28.68 ± 2.26^##^	6.69 ± 0.16^#^	6.27 ± 0.25^#^
**Baicalein**	2.61 ± 0.09^#^	3.47 ± 0.01	4.65 ± 0.25^###^	30.67 ± 1.99^##^	7.01 ± 0.36	6.30 ± 0.07^##^
**YSR**	2.73 ± 0.01^#^	3.69 ± 0.04	4.93 ± 0.41^##^	27.13 ± 1.58^##^	6.78 ± 0.16^#^	6.16 ± 0.019^##^
**WSR**	2.64 ± 0.09^#^	3.75 ± 0.164	4.31 ± 0.259^###^	33.14 ± 2.87^##^	6.94 ± 0.369	5.68 ± 0.494^##^

The data are presented as the mean ± S.D. (n = 7). *P < 0.05, **P < 0.01, ***P < 0.001 compared to the NC group; ^#^P < 0.05, ^##^P < 0.01, ^###^P < 0.001 compared to the self-healing group.

### 12 H Metabolism Quantity Test

Urine and stools were collected on the 4^th^ day after drug administration for assessment of 12 h metabolism quantity. In the self-healing group, stool weight was significantly increased while urine volume was significantly decreased (P < 0.05; **Table 2**). After treatment, compared with the model group, all treated rats showed reductions in stool volume, by varying degrees. There was also a significant increase in urine volume. The results show that the metabolism imbalance in the UC model was severe, while the metabolic ratios of the treatment groups were close to the normal group after 72 h. For all indices, baicalin was superior to baicalein, and YSR was superior to WSR, indicating that baicalin is more potent in regulating metabolism quantity.

**Table 2:** Metabolic of 12 h quantity in rats.

Group	Fecal weight (g)	Urine volume (ml)	F/U (g/ml)
**NC**	12.14 ± 0.25	5.77 ± 1.11	2.11 ± 0.51
**Self-healing control**	17.30 ± 0.02***	3.45 ± 1.26***	5.18 ± 0.68**
**SASP**	13.26 ± 1.12^#^	6.09 ± 1.65^##^	2.27 ± 0.13^##^
**Baicalin**	10.94 ± 2.53^###^	5.77 ± 1.19^##^	1.94 ± 1.48^##^
**Baicalein**	12.82 ± 1.51^###^	4.49 ± 0.92^###^	2.71 ± 1.22^##^
**YSR**	11.96 ± 2.17^###^	5.48 ± 1.41^###^	2.18 ± 1.77^##^
**WSR**	13.62 ± 2.44^##^	5.71 ± 1.01^###^	2.44 ± 1.84^##^

The data are presented as the mean ± S.D. (n = 7); **P < 0.01, ***P < 0.001 compared to the NC group; ^#^P < 0.05, ^##^P < 0.01, ^###^P < 0.001 compared to the self-healing group.

### Blood Biochemistry

**Figure 4:**
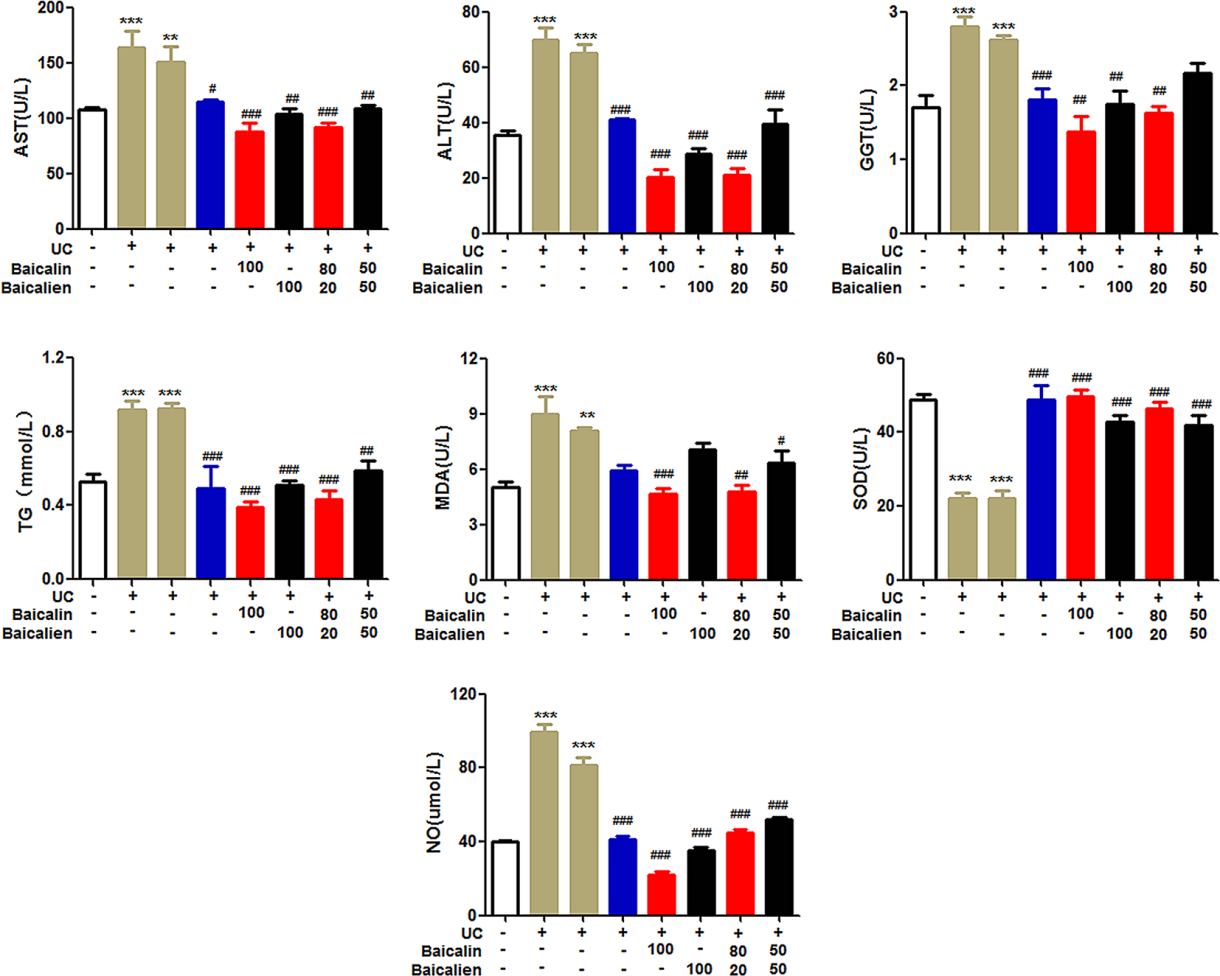
The levels of ALT, AST, GGT, NO, TG, MDA and SOD in rats. Data are presented as the mean ± S.D. (n = 7); ^**^P < 0.01, ^***^P < 0.001 compared to the NC group; ^#^P < 0.05, ^##^P < 0.01, ^###^P < 0.001 compared to the self-healing group. The left second column is UC model group, the left third column is self-healing group, and the left fourth column is SASP group.

As shown in **Figure 4**, ALT, AST, GGT, NO, TG, and MDA were significantly increased, and SOD was significantly decreased, in the model and self-healing groups compared to the NC group (P < 0.05). ALT, AST, GGT, NO, TG, and MDA were significantly higher (P < 0.05), and SOD was significantly lower, in the various treatment groups compared to the model and self-healing groups (P < 0.05). The baicalin group was lower than the other groups with the same drug dose. The results indicate that the UC model induced a certain degree of liver damage and caused elevated blood lipids, systemic inflammation, and oxidative stress. Baicalin was superior to baicalein at normalizing these blood indices, and YSR was superior to WSR, indicating that baicalin has more potency for regulating blood biochemistry.

### Energy Metabolism

**Figure 5:**
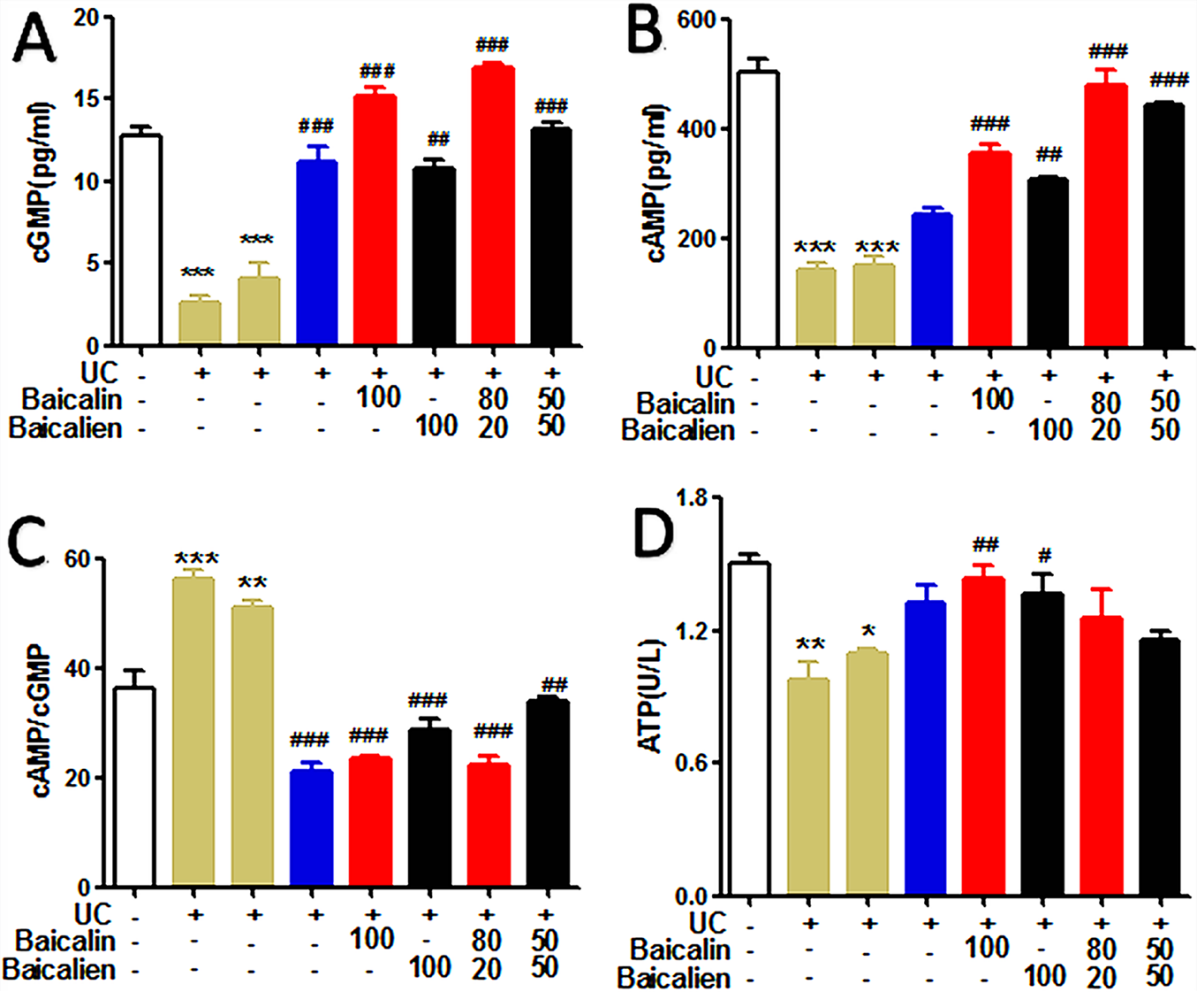
The levels of cAMP, cGMP, cAMP/cGMP and ATP in the rats. **(A)**, cGMP; **(B)**, cAMP; **(C)**, cAMP/cGMP; **(D)**, Na^+^K^–^ATP. The data are presented as the mean ± S.D. (n = 7); *P < 0.05, **P < 0.01, ***P < 0.001 compared to the NC group; ^#^P < 0.05, ^##^P < 0.01, ^###^P < 0.001 compared to the self-healing group. The left second column is UC group, the left third column is self-healing group, and the left fourth column is SASP group.

In order to investigate the therapeutic effects of baicalin, baicalein, and the combination of the two, on the energy metabolism of UC model rats, the levels of cAMP, cGMP, and cAMP/cGMP in serum and the levels of Na^+^K^+^-ATPase in liver tissue were determined. As shown in **Figure 5**, the levels of cAMP and cGMP in the serum of the model group and the self-healing group were significantly lower than those in the NC group (P < 0.01). Conversely, the level of cAMP/cGMP was higher than that of the NC group (P < 0.01). The levels of cAMP and cGMP in the various treatment groups were significantly increased compared to those in the model group and self-healing group (P < 0.01), but the cAMP/cGMP ratio was significantly lower (P < 0.01). The levels of Na^+^K^+^-ATPase in the liver tissues of the model group and self-healing group were significantly decreased compared to the NC group (P < 0.05) and were increased after the administration of each drug. The findings indicate that baicalin was superior to baicalein, and YSR was superior to WSR, suggesting that baicalin is a more potent regulator of energy metabolism.

### Effects of Drugs on UC Pathology

#### Weight of the Large Intestine and Small Intestine

**Figure 6:**
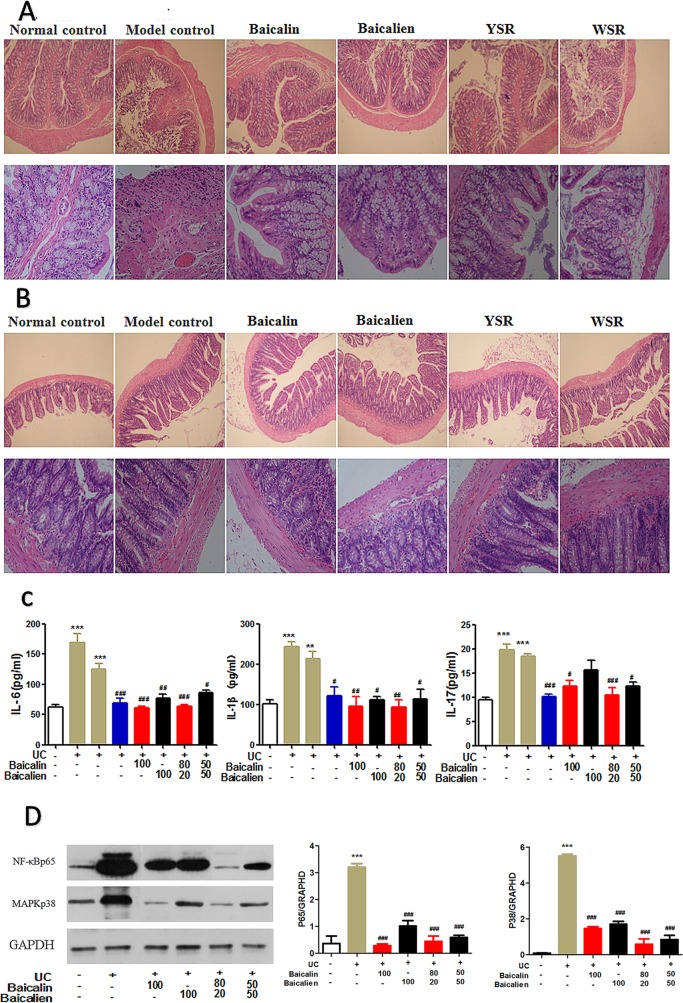
Protection effects of baicalin and baicalein in UC. **(A)**, Colon pathology; **(B)**, Ileum pathology; **(C)**, Inflammatory cytokines; **(D)**, NF-κB and MAPK protein. The data are presented as the mean ± S.D. (n = 7); **P < 0.01, ***P < 0.001 compared to the NC group; ^#^P < 0.05, ^##^P < 0.01, ^###^P < 0.001 compared to the self-healing group. The left second column is UC model group, the left third column is self-healing group, the left fourth column is SASP group.

As shown in **Table 3**, 7 days after drug administration, the large intestine and small intestine of the normal group were within the normal range while the large intestine and small intestine of the model group and the self-healing group were thick and fat with visible edema, congestion, and pustules. Compared with the control group, the weights of the large intestine and small intestine of the model group were significantly higher (P < 0.05). The weights of the large intestine and small intestine of each treatment group were significantly lower than those of the model group (P < 0.05). As shown in **Figures 6A, B**, there was severe tissue damage in the colon and small intestine of the UC model rats. Based on all indices of intestinal change, baicalin was superior to baicalein on the colon, and YSR was superior to WSR on the small intestine, indicating that baicalin is a more potent regular of colon damage.

**Table 3:** Measurement of the large intestine and small intestine.

Group	Small intestine (g)	Colon (g)	Colon length (cm)
**NC**	5.247 ± 0.016	2.221 ± 0.081	22.548 ± 2.548
**UC**	6.890 ± 0.167**	3.514 ± 0.146**	16.754 ± 1.541**
**Self-healing**	6.941 ± 0.165**	3.478 ± 0.159**	17.485 ± 2.742**
**SASP**	5.698 ± 0.017^##^	2.221 ± 0.335^##^	21.886 ± 1.872^##^
**Baicalin**	5.212 ± 0.168^##^	2.135 ± 0.215^##^	21.214 ± 1.911^##^
**Baicalein**	5.130 ± 0.199^##^	2.022 ± 0.217^##^	20.154 ± 2.348^##^
**YSR**	5.008 ± 0.184^##^	2.245 ± 0.304^##^	22.471 ± 1.374^##^
**WSR**	5.307 ± 0.265^##^	2.053 ± 0.191^##^	20.312 ± 1.147^##^

The data are presented as the mean ± S.D. (n=7); **P < 0.01, ***P < 0.001 compared to the NC group; ^##^P < 0.01, ^###^P < 0.001 compared to the self-healing group.

#### Histopathology Changes in the Colon and Ileum

In the NC group, the colonic mucosa of the rats was intact and the glands and lamina propria (LP) were arranged neatly without inflammatory cell infiltrations (**Figure 6A**). In the UC rats, the integrity of the colonic epithelium was severely disrupted, the glands were disordered or absent, and inflammatory cell infiltration, congestion, and edema were observed. The damage was notably ameliorated after treatment; the mucosal epithelium was regenerated, neutrophil infiltration decreased in the LP, and congestion and edema were effectively alleviated.

In the NC group, the mucosal epithelium of the ileum was intact, the morphologies of the villi were normal, and neither venous congestion nor inflammatory cell infiltration were observed in the LP. In the model group, inflammatory cells were observed in the LP and around the crypt, the integrity of the mucosal epithelium was severely destroyed, and some epithelium was exfoliated (**Figure 6B**). After treatment, the epithelium of the ileum was regenerated and intact, and there was a decrease in inflammatory cell infiltration.

### Effects on Inflammatory Cytokines

To study the anti-inflammatory effects and mechanisms of baicalin and baicalein in UC, we evaluated the levels of IL-6, IL-1β, and IL-17 in serum, as measured by ELISA (**Figure 6C**). As shown in **Figure 5**, the levels of IL-6, IL-1β, and IL-17 were significantly increased in the model and self-healing group compared to those in the NC group (P < 0.05). The levels of IL-17, IL-6, and IL-1β in the treatment groups were significantly decreased compared to those in the model and self-healing groups (P < 0.05). Overall, baicalin and baicalein inhibited UC by relieving the immuno-inflammatory response. Baicalin appeared to be superior to baicalein, and YSR was superior to WSR, indicating that baicalin is a more potent regulator of inflammation.

### Effect of Baicalin and Baicalein on the Nuclear Factor κB and MAPK Pathway

Activation of the nuclear factor-κB (NF-κB) and MAPK pathways increases the production of inflammatory cytokines and chemokines (**Figure 6D**). Compared with the NC group, NF-κB (STAT3) and MAPK (P38) protein expression in the UC group was significantly increased. After drug treatment, the expression levels of NF-κB and MAPK proteins were significantly decreased compared to those in the UC model group. Baicalin was found to exert a superior effect to baicalein, and YSR was superior to WSR, indicating that baicalin is a more potent regulator of inflammatory pathways.

### Drug Metabolism Detected by UPLC-MS/MS

**Figure 7:**
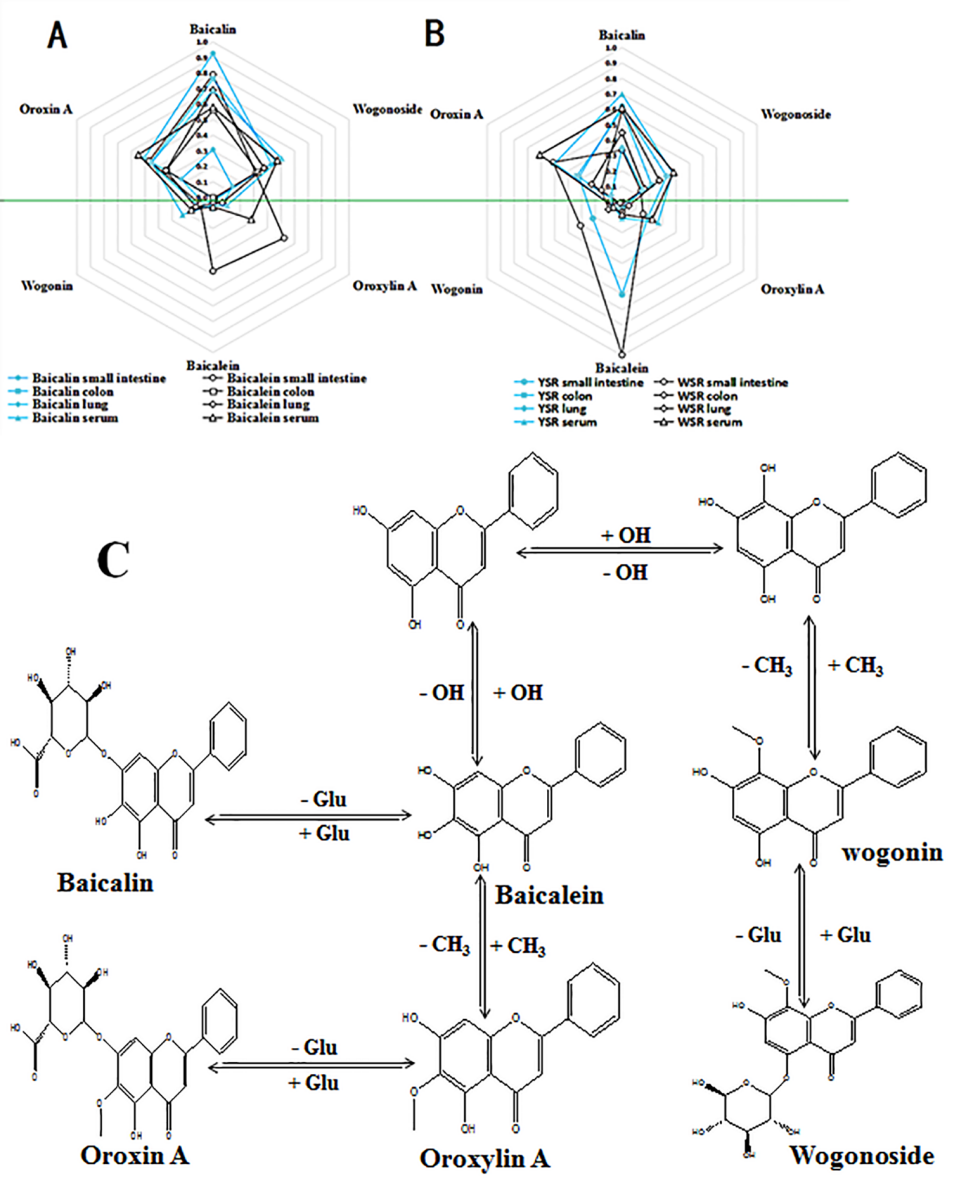
**(A)** Six metabolite levels of baicalin and baicalein group; **(B)** Six metabolite levels of YSR and WSR group; **(C)** Proposed metabolic pathways of the main active constituents after oral administration of baicalin and baicalein extracts to rats.

The peak area from the LC-MS/MS measurements was calculated (**Figure 7**). The six primary metabolites, baicalin, wogonoside, oroxin A, baicalein, wogonin, and oroxylin A, were identified in the small intestine, colon, lung tissue, and serum. The levels of the six metabolites were significantly different in the small intestine, colon, and lung tissue of each treatment group (P < 0.05), while there was no significant difference in serum (P > 0.05). When administered baicalin, baicalein, or the two drugs in combination, both baicalin and baicalein were generated and could be detected in the rat body and in the conversion pathway. Similarly, the four other metabolites, wogonoside, oroxin A, wogonin, and oroxylin A, were detected in the same regions, see **Figure 7** and **Table 4**.

**Table 4:** Mass spectrometric conditions for each compound.

Code	Compound Name	tR (min)	Ion mode	MRM (m/z)	Formula
1	Baicalin	2.341	ESI+	447.0→270.8	C_21_H_18_O_11_
2	Wogonoside	2.764	ESI+	461.0→284.8	C_22_H_20_O_11_
3	Oroxlin A	3.019	ESI+	461.0→284.8	C_22_H_20_O_11_
4	Baicalein	3.801	ESI+	271.0→253.0	C_15_H_10_O_5_
5	Wogonin	5.091	ESI+	285.0→270.0	C_16_H_12_O_5_
6	Oroxylin A	5.404	ESI+	285.0→270.0	C_16_H_12_O_5_

In the small intestine, the peak areas of baicalin, baicalin, and oroxin A in the baicalin group were significantly higher than the corresponding peak areas in the baicalein group, and the peak areas of these metabolites in the YSR group were also significantly higher in the WSR group. The peak areas of baicalein, wogonin, and oroxylin A in the baicalin group were significantly lower than those of the baicalein group, and the peak areas of the YSR group were also significantly lower than those of the WSR group (P > 0.05).

In the colon, the peak areas of the six metabolites in the baicalin group were significantly higher than those in the baicalein group, and the peak areas of the six metabolites in the YSR group were significantly higher than those in the WSR group (P > 0.05).

In the lungs, the peak areas of the six metabolites in the baicalin group were significantly lower than those in the baicalein group, and the peak areas of the six metabolites in the YSR group were significantly lower than those in the WSR group.

In serum, the peak areas of the six metabolites in the baicalin group were not significantly different from the baicalein group, and the peak areas of the six metabolites in the YSR group were not significantly different from those of the WSR group (P > 0.05). The results indicate that baicalin and baicalein, and the combination of the two, are distributed around the body in a certain manner.

## Discussion

We examined the therapeutic effects and mechanisms of action of baicalin and baicalein in the treatment of UC model rats. We found that the two drugs have significant therapeutic differences in terms of inhibition of increased body temperature, weight loss, inflammation, lipids, and liver disturbances. Further, the findings indicate that there are different mechanisms underlying the effects of each drug on inflammatory cytokines in UC. Our study shows that the basic treatment of intestinal diseases such as UC is through anti-inflammatory pathways. The mechanism of action is likely through regulation of the Th17/Treg balance and control of intestinal bacteria. Baicalin, more than baicalein, inhibits inflammatory factors IL-1β and IL-6 and activates the MAKP and NF-κB signaling pathways to induce IL-17 production. The antibacterial and antiviral effects of baicalin and baicalein underlie the treatment effects of these drugs in UC.

In this experiment, YSR treatment was found to be superior to the other treatments for UC. In a previous study, we found that YSR is the best overall treatment for UC model rats, and its effects were superior to sulfasalazine. As a result of the current study, we have shown that baicalin is superior to baicalein, and YSR is superior to WSR, indicating that baicalin is better at regulating the damage associated with UC in rats.

In this study we observed an obvious increase in body temperature, diarrhea, weight loss, edema, hyperlipidemia, and other symptoms in UC rats. Further, in UC model rats we found serum biochemical indicators of inflammation (NO) (Joh et al., 2011) and liver edema (ALT, AST, and GGT) (mei Jia et al., 2017), as well as altered blood biochemical indices (TG, SOD, MDA) (Awaisheh, 2016) and intestinal mucosal damage (Seyhan and Canseven, 2006). We also observed liver-protective effects (as deduced from changes in ALT, AST, and GGT) and blood fat adjustments (change of TG) associated with baicalin, baicalein, and the combination of the two drugs.

In the UC model, the generation of NO in the immune system can not only help resist the microbes invading the body, but can also prevent the proliferation of cancer cells and the spread of tumor cells. The results show that baicalin, baicalein, and the two drugs combined, can decrease NO content, indicating that these drugs aid the immune system of UC rats. The damage process associated with gastric ulcers may be related to peroxidative damage; in our study, each treatment group exhibited a decrease in SOD (intestinal mucosal attack factor) and an increase in MDA (intestinal mucosal protective factor (Patel, 2004). After treatment, the above indices all recovered to normal, but to a different extent in each group. The pathological changes in the inflammatory response in multiple organs were alleviated or returned to normal after treatment. Based on the anti-inflammatory, liver-protecting, hypolipidemic, and intestinal antioxidant stress effects, baicalin was found to be superior to baicalein, and YSR was superior to WSR, indicating that baicalin is a more potent regular of UC damage. This may be because baicalin has a long-retention time and slow absorption in the large intestine. This assertion is consistent with the traditional knowledge indicating that the high content of baicalin in Tiaoqin makes it better than Kuqin at treating disease of the large intestine, shown in **Table 5**.

**Table 5:** The comparative of the effects and mechanisms of baicalin, baicalein and their combinations on UC rat.

Group	Fever	inflammation	NF-κB (p65)	MAPK(p38)	Oxidative stress	liver damage	Colon damage	Small intestine damage	Lung indices	Brain indices	Yin-Yang insufficient
Baicalin	↓↓	↓↓	↓↓	↓↓	↓↓	↓↓	↓↓	↓	↓	↓	↑↑
Baicalelin	↓*	↓*	↓*	↓*	↓**	↓**	↓**	↓↓**	↓↓*	↓↓*	↑*
YSR	↓↓	↓↓	↓↓	↓↓	↓↓	↓↓	↓↓	↓↓	↓↓	↓↓	↑↑
WSR	↓^#^	↓^#^	↓^#^	↓^#^	↓^##^	↓^#^	↓^##^	↓↓^##^	↓^#^	↓^#^	↑^##^

↓ (decrease), ↑ (increase). The data are presented as the mean ± S.D. (n = 7). *P < 0.05, **P < 0.01, compared to the baicalin group; ^#^P < 0.05 ^##^P < 0.01, compared to the YSR group.

In the rat colitis model, IL-1β, IL-6, and IL-17 have been recognized as important proinflammatory cytokines in the course of UC, and overexpression of these cytokines is related to activation of the NF-κB and MAPK pathways in colonic tissue. A previous study found that paeoniflorin has an anti-inflammatory effect on UC *via* inhibition of the MAPK and NF-κB pathways and inhibition of apoptosis in rats (Pandurangan et al., 2015). The current results indicate that the NF-κB and MAPK pathways are partly involved in the therapeutic effects of baicalin and baicalein in UC. Previous reports have highlighted the role of probiotics (such as Lactobacillus rhamnosus GG and Lactobacillus casei) in modulating the intestinal immune system *via* the NF-κB and MAPKp38 signaling pathways (Joh et al., 2011). In the current study, we also found that T cell immune balance, particularly the balance of Treg/T17, plays a crucial role in the treatment of UC. Our study identified that the mechanism underlying treatment of UC with baicalin and baicalein involves inhibition of the increased levels of IL-6, IL-17, IL-1β, NF-κB, and MAPKp38; although, the inhibitory effects of the two drugs were significantly different. These findings have triggered a study of the differences in the anti-inflammatory effects of baicalin and baicalein mediated by the NF-κB and MAPK pathways in UC mice.

In this experiment, the UC rats exhibited daily diarrhea characterized by watery or bloody stools more than five times per day together with yellow urine, fever symptoms, and body temperatures exceeding 38.3°C. The gut is a complex anaerobic environment that is inhabited by a diverse gut microbial community and is affected by the long residence time of food. After *E. coli* infection, the cells of the intestinal epithelium exhibit apoptosis leading to reduced moisture regulation of the gut; this results in increased moisture in the feces and decreased urine (Lorber and Swenson, 1975). Examination of the weights of the large intestine and small intestine, the 12-h outputs of feces and urine, and the pathology of the ileum and colon, indicated that UC model rats exhibited pathology consistent with UC. Overall, we found that baicalin is best for treatment of the large intestine in UC rats, while baicalein is better for treatment of the small intestine.

UC is a complex and chronic disease with no clear treatment. In TCM, UC is considered to involve Yang deficiency of the spleen and kidney, and its main metabolic characteristics are loose stools and short yellow urine (Harvey et al., 1990). Goldbergnd and others believe that cAMP and cGMP are two opposing regulatory systems *in vivo* and these may be the material basis to explain the TCM Yin-Yang hypothesis (Lu, 2017). In this work, after treatment with baicalin, baicalein, and a combination of the two, cAMP, cGMP, cAMP/cGMP ratio, and ATPase were significantly increased compared to the UC model rats, which supports the Yin and Yang deficiency hypothesis of UC (Zhang et al., 2017). Further, the long-term fever that occurs in UC results in heat generation and a decrease in energy substances, leading to Yang insufficiency. Furthermore, diarrhea causes decreased body fluid and results in Yin insufficiency in the UC rats. The typical symptoms of Yang and Yin insufficiency are laziness, lethargy, and mental distraction. In the current study, after treatment, all of the UC symptoms in the rats were relieved. The treatments induced recovery of bodily functions and thus, recovery of the Yin and Yang insufficiency status of the UC rats.

The current study found significant differences in the *in vivo* effects of baicalin and baicalein in the digestive tract. Researchers have reported that baicalin is first converted into baicalein and oroxylin A under the action of bacteria in the large intestine cavity. They are then converted into baicalin and other substances by the enzymes in the intestine, and finally, enter the bloodstream. Researchers believe that the absorption of drugs in the intestine is related to drug polarity and local concentration in the tissue. Absorption is higher and faster for low polarity drugs compared to high polarity drugs. There is also a positive correlation with the local concentration of the drug. Therefore, we detected the drug concentrations in the lungs, small intestines, colons, and serum of each group using UPLC-MS/MS. We found that both baicalin and baicalein were converted into four new metabolites: wogonin, wogonoside, oroxylin A, and oroxin A. The concentrations of these metabolites were significantly different in the lungs, small intestine, and colon. All four metabolites were found to have anti-inflammatory activity. The content differences of the six metabolites may work together to produce differences in efficacy. This may explain why baicalin is effective for treating colon diseases while baicalein is effective for treating lung diseases. Furthermore, this may be the basis for the effects of Tiaoqin and Kuqin on the large intestine and lungs. The above results indicate that baicalin and baicalein have a differing distribution tendency, which is consistent with the traditional ideas on the use of young *Scutellaria baicalensis* (Tiaoqin in Chinese) and withered *Scutellaria baicalensis* (Kuqin in Chinese) in TCM.

In result, baicalin, baicalein, and the two drugs in combination, have significantly different in the anti-inflammatory, liver protection, lipid-lowering, anti-oxidation, and immunomodulation effects. Furthermore, there are significant differences in the drug-induced expression of the NF-κB and MAPK signaling pathways. These differences may cause the differences in the distribution of metabolites.

## Conclusion

*Scutellaria baicalensis* is a herbal medicine traditionally used for the treatment of UC. The main components of *Scutellaria baicalensis* are baicalin and baicalein. In this study, we tested each component separately and in combination for the treatment of UC. Baicalin, baicalein, and a combination of the two drugs, had significantly different effects on UC. We found that baicalin and baicalein have different advantages in treating UC, and a combination of the two drugs provides more comprehensive treatment; specifically, YSR was the most effective treatment. The proportion of baicalin in YSR is higher than the proportion of baicalein, consistent with the natural proportion of baicalin and baicalein in young *Scutellaria baicalensis*. This study compared the mechanisms of action of baicalin and baicalein based on pharmacodynamic indicators and evaluation of chemical composition using metabolomics techniques, UPLC-MS/MS, and traditional pharmacological indicators. It was found that compared with baicalein, baicalin was more potent for the treatment of large intestine disease. The mechanism underlying its effects may be related to the slower absorption of its metabolites in the large intensity as compared to baicalein. This increased retention time may aid in regulating UC. These results indicate that baicalin and baicalein should be used to target more specific diseases and may prove useful as new alternatives or supplements for clinical drug development.

## Data Availability Statement

All datasets generated for this study are included in the article/**Supplementary Material**.

## Ethics Statement

The animal study was reviewed and approved by the Animal Ethics Committee and Institutional Animal Care and Use Committee of South-Central University for Nationalities.

## Author Contributions

SL, XD, YZ, JA, LL, LC, HX and YR participated in the experiments. SL, XD and YR analyzed the data and wrote the paper. YR secured the funding, designed the experiments, and revised the paper. LC, ZM, Y-CC, and YR provided technical guidance. SL, YZ, XD, JA, HX and LL participated in experimental preparation and statistical analysis. All authors read and approved the final manuscript.

## Funding

This work was supported by National Natural Science Foundation of China grants (No. 81773893, No. 31600272 and No.81503203), National Major Scientific and Technological Special Project for “Significant New Drugs Development” (No. 2017ZX09301060-001), National Key R&D Program of China (No.2017YFC171004), Hubei Province Natural Science Foundation of China (No. 2015CFB302), and Fundamental Research Funds for the Central Universities “South-Central University for Nationalities” (No. CZP17074).

## Conflict of Interest

The authors declare that the research was conducted in the absence of any commercial or financial relationships that could be construed as a potential conflict of interest.
