# Supplementary material for: The Comparative Study of the Therapeutic Effects and Mechanism of Baicalin, Baicalein, and Their Combination on Ulcerative Colitis Rat
Source: Front Pharmacol. 2019 Dec 13;10:1466. doi: 10.3389/fphar.2019.01466 (PMC6923254; doi:10.3389/fphar.2019.01466)
Supplement: Supplementary file 1 [file DataSheet_1.docx]

**The content of YSR and WSR determination**

# Sample preparation:

Tiaoqin and Kuqin sample was identified as Tiaoqin and Kuqin,the root of *Scutellaria baicalensis* Georgi by Professor Liao Maochuan of School of Pharmacy of South-Central University for Nationalities.

Firstly, the medicinal materials (50 g) were mixed and soaked for 30 min with 10 times (v/w) distilled water and then decocted for 120 min; secondly, the filtrates were collected and the residue was decocted again for 60 min with six times (v/w) distilled water; finally, the filtrates were mixed and condensed to the required volume (0.1 g/ml) by rotary evaporation at 60 °C.

Tiaoqin (Shaanxi Medicinal Material Market), concentration: 0.1 g/mL; Kuqin (Shaanxi Medicinal Material Market), concentration: 0.1 g/mL.

# HPLC condition：

Column: Thermo BDS HYPERSIL C_18_ Column (4.6 mm × 250 mm, 5μm), A flow rate of 1.0 mL∙min^-1^ and a column temperature of 30 °C, the injection volume was 15 μL, detection wavelength: 274 nm.

The mobile phase consisted of (A) acetonitrile and(B) was water(containing 0.1% formic acid). The step gradient program was as follows:：0~30 min，10%~25% (A)；30~60 min, 25%~45% (A), 60~65 min, 45%~90%(A).

# HPLC chromatogram comparison

As shown in Supplementary Figures 1 and Supplementary Table S1. The concentration of baicalin and baicalein in the drug sample was converted to comparison with the standard of baicalin and baicalein. We found that the content of baicalin: baicalein = 4 : 1 in Tiaoqin (YSR), and the content of baicalin : baicalein = 1 : 1 in Kuqin (WSR).


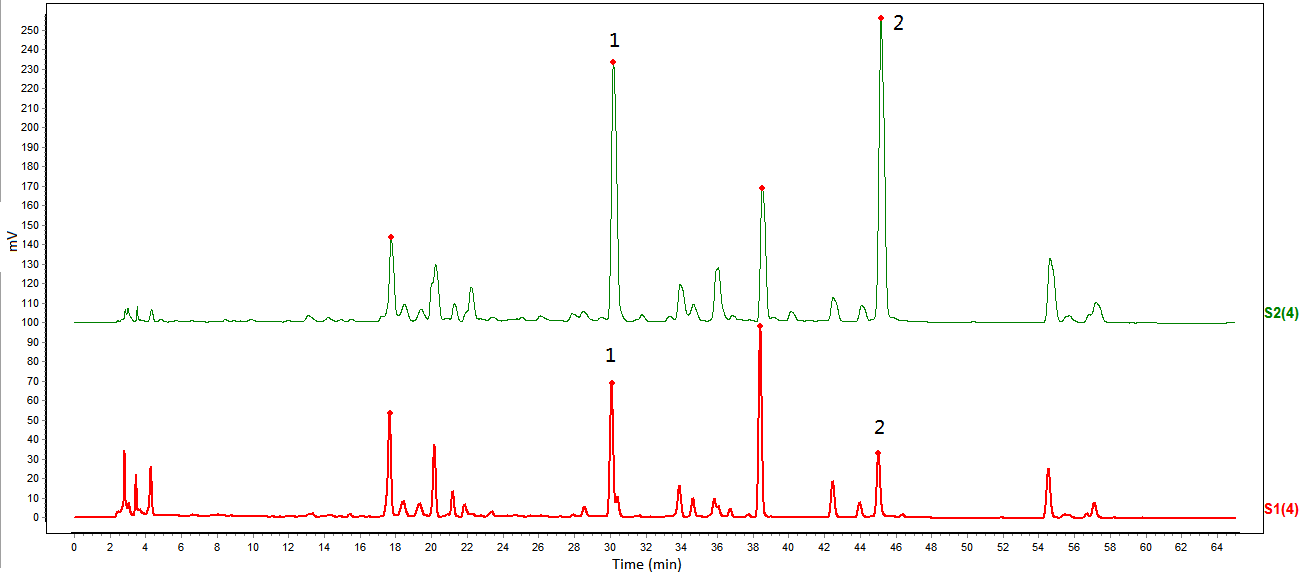
Supplementary Figure 1. the HPLC result of Tiaoqin and Kuqin. Tiaoqin(S1), Kuqin(S2), baicalin (1), baicalein (2).

Supplementary Table S1. the content of baicalin and baicalein in Tiaoqin and Kuqin.

| Drug sample | Baicalin(mg/ml) | Baicalein(mg/ml) | Baicalin:baicalein |
| --- | --- | --- | --- |
| Tiaoqin(0.1g/ml) | 9.63 | 2.62 | 4:1(YSR) |
| Kuqin(0.1 g/ml) | 30.12 | 31.67 | 1:1(WSR) |

# Conclusion

From the results of HPLC of Tiaoqin (0.1 g/ml) and Kuqin (0.1 g/mL), we can conclude that the concentration ratios of baicalin and baicalein in Tiaoqin (0.1 g/mL) in Tiaoqin was averagely 4:1 (YSR) , and the concentration ratios of baicalin and baicalein in Kuqin was averagely 1:1 (WSR). Therefore, we used the ratio of YSR and WSR to study the pharmacodynamics and mechanism differences of the combination of baicalin and baicalein in UC model rats.
